# New Medical Works

**Published:** 1867-12

**Authors:** 


### New Medical Works.

Among the new announcements of Medical Works shortly to appear, we notice that Messrs. Lindsay & Blackisten, of Philadelphia, are preparing a volume of Surgical Reports and Clinical Lectures, by the Medical and Surgical Staff of the Pennsylvania Hospital. The reputation of this institution, now in the second century of its existence, the corner stone having been laid by Benjamin Franklin, warrants us in anticipating a very valuable and interesting volume. It will be brought out upon the plan of "Guy's" and "St. Barthelomew's Hospital Reports," published so successfully for many years in London. The first voiume will contain an introductory article by Prof. Meigs, formerly connected with the hospital, giving many interesting remeniscences of its early history, and the physicians who have served it. The articles will be illustrated when necessary, and the work will be continued from year to year.

The same publishers will also issue early in January, an Annual of Therapeutics Pharmacology, &c., &c., by A. Bourchadat, Professor of Hygiene, &c.. to the Faculty of Medicine, Paris, translated and edited in this country by M. J. De Rosset, M. D., Adjunct to the Professor of Chemistry University of Maryland. The eminently practical character of this volume, which has now been published for twenty-eight successive years in Paris, with a large circulation all over the continent, warrants the supposition that it will meet with a very favorable reception in the United States.

